# COX-2 Inhibition Reduces *Brucella* Bacterial Burden in Draining Lymph Nodes

**DOI:** 10.3389/fmicb.2016.01987

**Published:** 2016-12-12

**Authors:** Aurélie Gagnaire, Laurent Gorvel, Alexia Papadopoulos, Kristine Von Bargen, Jean-Louis Mège, Jean-Pierre Gorvel

**Affiliations:** ^1^Aix Marseille Univ, CNRS, INSERM, CIML, Centre d’Immunologie de Marseille-LuminyMarseille, France; ^2^Department of Pathology and Immunology, Washington University School of Medicine, St. LouisMO, USA; ^3^Aix Marseille Univ, INSERM, CNRS, IRD, URMITEMarseille, France

**Keywords:** *Brucella*, COX-2, dendritic cells, intradermal infection, prostaglandins

## Abstract

*Brucella* is a Gram-negative facultative intracellular bacterium responsible for a chronic disease known as brucellosis, the most widespread re-emerging zoonosis worldwide. Establishment of a Th1-mediated immune response characterized by the production of IL-12 and IFNγ is essential to control the disease. Leukotrienes derived from arachidonic acid (AA) metabolism are known to negatively regulate a protective Th1 immune response against bacterial infections. Here, using genomics approaches we demonstrate that *Brucella abortus* strongly stimulates the prostaglandin (PG) pathway in dendritic cells (DC). We also show an induction of AA production by infected cells. This correlates with the expression of *Ptgs2*, a gene encoding the downstream cyclooxygenase-2 (COX-2) enzyme in infected DC. By comparing different infection routes (oral, intradermal, intranasal and conjunctival), we identified the intradermal inoculation route as the more potent in inducing *Ptgs2* expression but also in inducing a local inflammatory response in the draining cervical lymph nodes (CLN). NS-398, a specific inhibitor of COX-2 enzymatic activity decreased *B. melitensis* burden in the CLN after intradermal infection. This effect was accompanied by a decrease of *Il10* and a concomitant increase of *Ifng* expression. Altogether, these results suggest that *Brucella* has evolved to take advantage of the PG pathway in the harsh environment of the CLN in order to persist and subvert immune responses. This work also proposes that novel strategies to control brucellosis may include the use of COX-2 inhibitors.

## Introduction

Brucellosis is a disease caused by a Gram-negative facultative intracellular bacterium belonging to the genus *Brucella.* It is the most widespread re-emerging zoonosis worldwide affecting more than half a million people each year (Seleem et al., 2010). *Brucella* affects a wide range of land and aquatic mammals including humans and livestock. Animals are *Brucella* primary hosts in which the bacterium has a particular tropism for the reproductive system, often leading to spontaneous abortion and sterility responsible for severe economic losses (Pappas, 2010). *Brucella* can be transmitted to humans in close contact with infected animals mostly by ingestion of contaminated food or inhalation of aerosolized contaminated particles (Moreno, 2014). The *Brucella* species presenting the most important zoonotic potential for humans are *B. melitensis*, *B. abortus*, *B. suis*, and *B. canis* (Moreno, 2014). In human, the disease is characterized by an acute phase, which mainly manifests itself by asthenia, recurrent undulant fever and influenza-like symptoms. If the disease is not cured, it can evolve into chronic brucellosis, which may result in serious complications such as endocarditis or neurobrucellosis (Franco et al., 2007; Dean et al., 2012). Currently, there is no effective vaccine to prevent the disease in human highlighting the importance to understand the physiopathology of the disease.

To establish a persistent infection, *Brucella* behaves as a stealthy pathogen (Barquero-Calvo et al., 2007), using various strategies to modulate the host immune response. For instance, its unusual LPS plays a central role showing a low endotoxicity and being poorly recognized by Toll-like-receptor 4 (TLR4) (Lapaque et al., 2005; Lapaque et al., 2009; Conde-Álvarez et al., 2012). *Brucella* can also modulate the protective Th1 immune response characterized by the secretion of pro-inflammatory cytokines such as IL-1β, IFNγ, TNF-α, or IL-12 (Zhan and Cheers, 1995; Zhan et al., 1996; Martirosyan et al., 2011) by inducing an early IL-10 secretion orchestrated by CD4^+^CD25^+^ T cells (Xavier et al., 2013).

**FIGURE 1:**
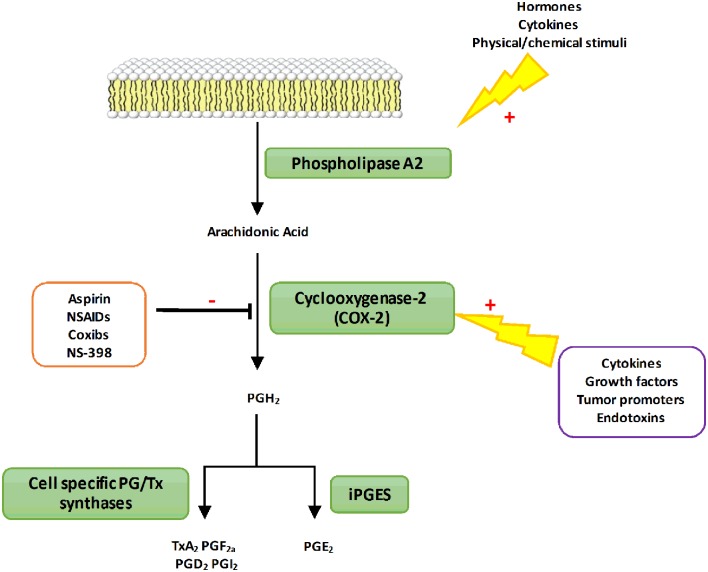
**The PGs biosynthetic pathway**. In response to different types of stimuli (hormones or cytokines), phospholipase A2 is activated to produce AA from membrane phospholipids. Free AA can be metabolized by the COX-2 enzyme to produce PGs H_2_ (PGH_2_). PGH_2_ is then converted into prostanoids through the action of various synthases differentially expressed in cells. PG, Prostaglandins; TX, Thromboxane; NSAIDs, Nonsteroidal anti-inflammatory drugs; COX-2, Cyclooxygenase-2; iPGES, inducible Prostaglandin synthase; TxA_2_, Thromboxane A_2_; PGF_2α_, Prostaglandin F_2α_; PGI_2_, Prostaglandin I_2_; PGE_2_, Prostaglandin E_2_.

It has also been shown that *B. abortus* can activate the 5-LO responsible for the synthesis of leukotrienes derived from AA (Fahel et al., 2015). The 5-LO activity impacts on pro-inflammatory cytokine secretion including IL-12 and IFNγ *in vivo* (Fahel et al., 2015). Thus, 5-LO seems to function as a negative regulator of the protective Th1 response during mice infection with *B. abortus*. However, AA can be metabolized by different enzymes including the COX also known as Ptgs (Harizi et al., 2008). COX enzymes are responsible for the rate-limiting step in the biosynthesis of PG and other prostanoids among them thromboxanes and prostacyclins (**Figure 1**). Three known isoforms of COX have been identified so far: COX-1, COX-2 and COX-3 whose physiological function is not yet fully elucidated (Chandrasekharan et al., 2002; Chandrasekharan and Simmons, 2004). The constitutively and ubiquitously expressed COX-1 isoform is involved in basal and constitutive PG production. On the contrary, COX-2 is expressed at very low levels and is highly inducible following pro-inflammatory stimuli such as cytokines or endotoxins. COX-2 converts AA into prostaglandin H_2_ (PGH_2_), which is then converted into prostaglandin D_2_, prostaglandin E_2_ (PGE_2_), prostacyclins, or thromboxane A_2_ depending on cell specific enzymes expression (**Figure 1**). The context as well as the panel of PG receptors will define the pro- or anti-inflammatory action of these lipid mediators. Thus, PG have immunomodulatory traits that can worsen or improve bacterial clearance. This dual activity is observed during *Salmonella* Typhimurium infection in mice. Indeed, COX-2 inhibition during the acute stage of the disease increases bacterial load whereas at later stage it enhances mouse survival (Bowman and Bost, 2004). A similar pattern is observed in mice infected with *Mycobacterium tuberculosis* (Rangel Moreno et al., 2002).

Cyclooxygenase-2 has been shown to have different outcomes during several bacterial and viral infections (Steer and Corbett, 2003; Agard et al., 2013) but little is known about its role during *Brucella* infection. Some studies have reported the impact of *B. abortus* and *B. melitensis* LPS on COX-2 expression in monocytes (López-Urrutia et al., 2001). Both smooth-LPS (S-LPS) were found to induce COX-2 expression in THP-1 cell line and rat-peritoneal macrophages in a dose-dependent manner. PGE_2_ synthesis and its impact in bovine PBMC infected with live and γ-irradiated *B. abortus* have also been assessed showing that IL-2 and IFNγ suppression was independent on COX-1 and COX-2 (Stevens and Olsen, 1994) but these *in vitro* studies were not confirmed *in vivo*.

The aim of our study is to determine whether COX-2 is involved in protection against *Brucella* infection or exacerbation of the *Brucella* burden. We have investigated the ability of *B. abortus* to stimulate the AA-COX-2 pathway in infected human moDC and in mouse GM-CSF BMDC and studied the impact of COX-2 inhibition *in vivo* after intradermal infection with *B. melitensis*. Here, we demonstrate that *B. abortus* enhances AA production in human moDC and *Ptgs2* expression both in human moDC and murine GM-CSF BMDC. The comparison of different inoculation routes allowed us to identify the intradermal route as the more potent to induce *Ptgs2* expression and local inflammatory response in CLN. COX-2 inhibition decreases the bacterial burden in CLN characterized by a decrease of *Il10* and an increase of *Ifng* expression. Thus, further development of COX-2 inhibitors may represent a novel therapeutic strategy against *Brucella*.

## Materials and Methods

### Ethics Statement

This study was carried out in strict accordance with the guidelines of the Council of the European Union (Directive 86/609/EEC) regarding the protection of animals used for experimental and other scientific purposes. The experimental protocol was approved by the institutional animal care and use committee of the Aix-Marseille University (N° 87–848, October 19, 1987, Paris, modified by Decree 2001-464 and Decree 2001-131 relative to European Convention, EEC directive 86/609). All experiments were done in accordance with French and European guidelines for animal care. Blood was obtained from the Etablissement Français du Sang. As it is a commercial product, the Ethics Committee agreement is not required.

### Mice and Bacteria

Six to eight weeks old females C57BL/6 (Charles River Laboratories) were maintained under pathogen-free conditions and fed sterile food and water *ad libitum.* All infections were performed under Animal Biosafety Level 3 facility.

### Bacterial Strains

*Orentia tsutsugamushi* strain Kato (CSUR R163) (gift from Dr. Jean-Louis Mège, URMITE, Marseille, France) was propagated in L929 cells (ATCC), as previously described (Barry et al., 2012). Briefly, highly infected cells were sonicated, or lysed using glass beads for *O. tsutsugamushi*, and centrifuged at 500 × *g* for 10 min to remove cell debris. Supernatants were collected and centrifuged at 10,000 × *g* for 10 min to pellet the bacteria. Live bacteria were quantified using infected cell-counting units. *Coxiella burnetii* organisms (RSA493 Nile Mile strain) (gift from Dr. Jean-Louis Mège, URMITE, Marseille, France) were obtained by passage in BALB/c mice (Charles River Laboratories) and culture in L929 cells. The concentration of organisms was determined by immunofluorescence using specific antibodies and/or PCR using known concentrations of bacterial DNA. The quantification of organisms was performed as previously described (Barry et al., 2012). Bacterial viability was assessed using the LIVE/DEAD Bac Light bacterial viability kit (Invitrogen). *Brucella abortus* strain 2308 and *Brucella melitensis* strain 16M (gift from Dr Ignacio Moriyon, University of Pamplona, Spain) were grown on TSA (Sigma-Aldrich) at 37°C for 7 days, as previously described (Pizarro-Cerdá et al., 1998). The Twist-Marseille (CNCM I-2202) strain of *Tropheryma whipplei* (gift from Dr. Jean-Louis Mège, URMITE, Marseille, France), a bacterium known to live in macrophages, was cultured in HEL cells and purified. Quantification of inocula was performed by measuring the percentage of infected cells by immunofluorescence, as previously described (Gorvel et al., 2010).

### Isolation of Human moDC

Peripheral blood mononuclear cells from buffy coats were recovered from the Ficoll-Hypaque (GE healthcare) interface after centrifugation at 700 × *g* for 20 min. Monocytes and T lymphocytes were isolated from PBMCs using magnetic beads coupled to antibodies specific for CD14 and CD3, respectively, as described by the manufacturer (Miltenyi Biotec). The purity of the monocyte and T-cell preparations was assessed by flow cytometry and was greater than 98%. Monocytes were then incubated in RPMI 1640 (Gibco) containing 20 mM HEPES, 2 mM glutamine, 10% fetal calf serum (FCS) (Invitrogen), 0.1 ng/mL IL-4 and 1 ng/mL granulocyte-macrophage colony-stimulating factor (GM-CSF) (R&D Systems) for 7 days to obtain moDC.

### *In vitro* Generation of BMDC

Bone marrow-derived dendritic cells were prepared from 6 to 8 week-old females C57BL/6 mice as previously described (Salcedo et al., 2008). Briefly, tibias and femurs were flushed with RPMI-5%FCS (Eurobio)-50 μM 2β-mercaptoethanol (Sigma-Aldrich) to extract bone marrow.

In order to generate GM-CSF-BMDC, 3.10^6^ cells per well were seeded onto 6-well plates in medium supplemented with supernatant of J558L GM-CSF producing cell line (gift from Dr. Philippe Pierre, Centre d’Immunologie de Marseille-Luminy, Marseille, France). The medium was changed at day 2.5.

### *In vitro* Infection Assays

GM-CSF-DC were infected with *B. abortus* smooth virulent strain 2308 using a MOI of 30:1. Bacteria were centrifuged onto cells at 400 × *g* for 10 min at 4°C. Then, cells were incubated for 30 min at 37°C with 5% CO_2_. After two washes with medium, cells were incubated for one hour with medium containing antibiotic [gentamicin (Sigma-Aldrich) at 50 μg/ml] in order to kill the extracellular bacteria. Thereafter, the antibiotic concentration was decreased at 10 μg/ml until the end of the infection time.

### *In Vivo* Bacterial Challenge

*Brucella melitensis* 16M with a chromosomal integration of DsRed was used in this study. *B. melitensis* were grown under shaking in TSB (Sigma-Aldrich) containing 25 μg/ml kanamycin (Sigma-Aldrich) at 200 rpm for 16 h at 37°C. Inocula were prepared by pelleting bacteria and by adjusting bacterial concentration in endotoxin free PBS (Gibco) to obtain the right concentration depending on the inoculation route. Oral infection was performed as previously described (von Bargen et al., 2014). Briefly, pelleted bacteria were re-suspended to previously heat-sterilized 5% milk and adjusted at respective MOI in 5% milk (Scharlau). For all the other inoculation routes, mice were anesthetized with Ketamine/Xylazine (Virbac). For intranasal infection, the bacterial inoculum containing 10^5^ CFU suspended in 35 μL of endotoxin free PBS (Gibco) was applied onto mice nostril via pipet. For intradermal infection, the bacterial inoculum containing 10^4^ CFU suspended in 10 μL of endotoxin free PBS was injected into the ear pinna. For conjunctival infection, the bacterial inoculum containing 10^9^ CFU suspended in 10 μL was applied onto conjunctival surfaces (5 μL per eye). In some experiments, the selective COX-2 inhibitor NS-398 (Cayman Chemicals) were administered by intraperitoneal injection (15 mg/kg) right after intradermal infection. A stock solution of NS-398 was prepared in DMSO (Sigma-Aldrich) and was diluted in PBS just prior to treatment to the right concentration in a 1:3 (DMSO:PBS) ratio for a total volume of 100 μL per mouse or vehicle control (DMSO:PBS alone).

Treatments were repeated daily for 7 days. At 8 and 29 days post-infection mice were sacrificed by cervical dislocation. Immediately after sacrifice, CLN (including mandibular, accessory mandibular, and superficial parotid lymph nodes referred here as CLN; Van den Broeck et al., 2006) and spleens were collected, weighted, and proceeded for bacterial count and RNA extraction.

### Analysis of Organ Bacterial Burden

Spleens and CLN were removed and homogenized in Triton X-100 (Sigma-Aldrich) 0.1%. Serial dilutions were performed in PBS, and plated in triplicate onto TSA agar. CFU were enumerated after 3-4 days of culturing at 37°C.

### Microarrays

Monocyte derived dendritic cells (5.10^6^ cells per well) were plated in 6-well plates and stimulated or not with *B. abortus* (MOI of 300:1) for 6 hours, and total RNA was then extracted using the RNeasy minikit (Qiagen) and DNase treatment. This study utilized the 4X44k Human Whole Genome microarrays (Agilent Technologies), representing 44,000 probes. Reverse transcription, samples labeling, and hybridization were performed according to the protocols specified by the manufacturer (One-Color Microarray-Based Gene Expression Analysis). Three samples per experimental condition were included in the analysis. The slides were scanned at a 2-mm resolution with a G2505C DNA microarray scanner (Agilent Technologies).

### RNA Extraction, RT, and q-PCR

Total RNAs from infected GM-CSF-BMDC were extracted using RNeasy Micro Kit (Qiagen) following manufacturer’s instructions. CLN were stabilized in RNAlater (Qiagen) immediately after sampling. Organs were homogenized in RLT buffer (Qiagen) and then extracted using RNeasy Micro Kit (Qiagen) following manufacturer’s instructions. cDNAs were obtained by using Quantitech Reverse Transcription Kit (Qiagen) following manufacturer’s instructions using 300 ng of RNA as a matrix. qPCR were conducted using a 7500 Fast-Real-time PCR (Applied Biosystem) with SYBER Green (Takara) following manufacturer’s instructions. HPRT was used as housekeeping gene to determine ΔCt. Fold change compared to base line expression in uninfected cells, or control mice was determined using 2^-ΔΔCt^ method where ΔΔCt = (Ct_target_-Ct_HPRT_)_infected_-(Ct_target_-Ct_HPRT_)_non-infected_ as previously described (Papadopoulos et al., 2016). mRNAs whose expression level was twice as high compared to control were considered as significantly up-regulated. Primers used in this study are listed in **Table 1**.

**Table 1:** Primers used for analysis of gene expression upon infection.

Genes	Forward Primers	Reverse Primers
*HPRT*	AGCCCTCTGTGTGCTCAAGG	CTGATAAAATCTACAGTCATAGGAATGGA


*Ifng*	TCAAGTGGCATAGATGTGGAAGAA	TGGCTCTGCAGGATTTTCATG


*Il6*	GAGGATACCACTCCCAACAGACC	AAGTGCATCATCGTTGTTCATACA


*Gzmb*	ATCAAGGATCAGCAGCCTGA	CATGATGTCATTGGAGAATGTCT


*Nos2*	CAGCTGGGCTGTACAAACCTT	CATTGGAAGTGAAGCGTTTCG


*Ptgs2 (Cox2)*	ACCTCTGCGATGCTCTTCC	TCATACATTCCCCACGGTTT


*Ccl2*	GCCTGCTGTTCACAGTTGC	ATTGGGATCATCTTGCTGGT


*Ccr7*	GTGGTGGCTCTCCTTGTCAT	GAAGCACACCGACTCGTACA


*Il10*	GGTTGCCAAGCCTTATCGGA	ACCTGCTCCACTGCCTTGCT


*Foxp3*	AGGAGCCGCAAGCTAAAAGC	TGCCTTCGTGCCCACTGT
*Tnf*	CATCTTCTCAAAATTCGAGTGACAA	TGGGAGTAGACAAGGTACAACCC
*Il4*	ACTCTTTCGGGCTTTTCGAT	TTGCATGATGCTCTTTAGGC

### Arachidonic Acid Quantification

Monocyte derived dendritic cells were stimulated by bacterial pathogens (MOI 50:1 for *T. whipplei* or 20:1 for others) or *Escherichia coli* LPS (Sigma-Aldrich) (1 μg/ml) for 16 h, suspended in 0.3 mL of cold methanol (MeOH). Fatty acid methyl esters (FAME) extraction was performed as previously described (Schutter and Dick, 2000; Ecker et al., 2012), 15 μL of MeOH/Deuterium-labeled eicosanoids (LTB4-d4 and 5-HETE-d8, 400 ng/ml) standard solution were added to 0.2 ml of homogenate. After centrifugation, supernatants were diluted in 10 mL of hydrochloric acid (HCl) 0.02 M and submitted to solid-phase extraction on C18 cartridge (Macherey Nagel). After complete loading, columns were washed, dried and lipid mediators were eluted in methyl formate (MeFor). Solvent was evaporated under N2 and samples were dissolved within 30 μL MeOH and stored at -80°C.

For AA quantification, 0.1 ml of homogenates were extracted in dichloromethane/methanol/water, in the presence of 2 μg glyceryl triheptadecanoate (Sigma-Aldrich) and hydrolyzed in potassium hydroxyde (KOH) (0.5 M in MeOH) at 50°C for 30 min. Lipids were transmethylated in 14% boron trifluoride MeOH solution (Sigma-Aldrich) and hexane at 80°C for 1 h. FAME were extracted with hexane, dried and dissolved in ethyl acetate.

Fatty acid methyl esters were analyzed by gas-liquid chromatography as previously described (Lepage and Roy, 1986) on a Clarus 600 Perkin Elmer system using Famewax RESTEK fused silica capillary columns (30 m × 0.32 mm i.d, 0.25 μm film thickness). Oven temperature was programmed from 110°C to 220°C at a rate of 2°C per min and the carrier gas was hydrogen (0.5 bar). The injector and the detector were at 225°C and 245°C, respectively. LC-MS/MS analysis was performed on UHPLC system (Agilent LC1290 Infinity) coupled to Agilent 6460 triple quadrupole MS (Agilent Technologies) equipped with electrospray ionization source. Separation was done at 40°C on a Zorbax SB-C18 column (2.1 mm, 50 mm, 1.8 μm) (Agilent Technologies). The mobile phases were, respectively water, ACN and FA (75/25/0.1) (A) and ACN, FA (100/0.1) (B). The linear gradient was as follows: 0% B at 0 min, 85% B at 8.5 min, 100% B at 9.5 min, 100% B at 10.5 min, and 0% B at 12 min. The flow rate was 0.35 mL/min. The autosampler was set at 5°C and the injection volume was 5 μL. Electrospray ionization was performed in negative ion mode with a spray voltage fixed at -3500 V. After optimization, source conditions were as follows: source temperature was 325°C, nebulizer gas (nitrogen) flow rate was 10 L/min, sheath gas temperature was 400°C and sheath gas (nitrogen) flow rate was 12 L/min. Analyses were acquired in multiple reaction mode (MRM) using nitrogen as collision gas. For each compound the conditions of separation: retention time in minute (RT), and of quantification were defined: specific Q1/Q3 transition (T) fragmentor (F), and collision energy (CE). Thus, optimized parameters were as follows: LTB4 (RT: 4.32 min, T: 335/195, F: 115 V; CE: 4 V), LTB4-d4 (RT: 4.31 min, T: 339/197, F: 120 V, CE: 6 V). Peak detection, integration and quantitative analysis were performed using Mass Hunter Quantitative analysis software (Agilent Technologies).

### Statistical Analysis

The results are expressed as mean ± SD and statistical significance was assessed using the unpaired, two-tailed Student’s *t*-test or by analysis of variance (ANOVA) followed by Tukey’s multiple comparison test provided by GraphPad Prism software when more than two conditions were analyzed simultaneously. *p* values over 0.05 were not considered as significant.

## Results

### *B. abortus* Infection of moDC and GMCSF-BMDC Stimulates AA-Cyclooxygenase Pathway

**FIGURE 2:**
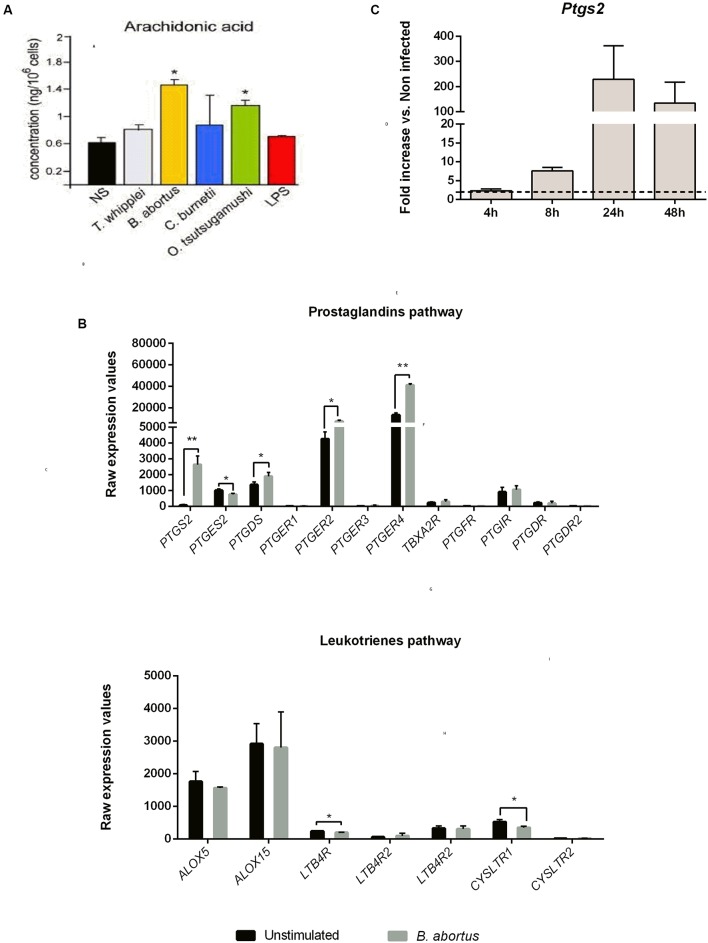
**Prostaglandins pathway highlights induced AA metabolism in human moDC and mice GM-CSF BMDC upon *Brucella abortus* infection**. **(A)** moDC were stimulated or not (NS) during 16 h with *Tropheryma whipplei* (30:1), *B. abortus* (20:1), *Coxiella burnetii* (20:1), *Orentia tsutsugamushi* (20:1), or *Escherichia coli* LPS (1 μg/mL) and then treated for AA dosage. The AA concentration after infection was compared to unstimulated condition using unpaired, two-tail Student’s *t*-test (^∗^*p* < 0.05) (Results of three independent experiments). Results are given as mean ± SD. **(B)** Transcriptomic analysis. moDC were stimulated or not with *B. abortus*, for 6 h. RNA was extracted, and a microarray was performed. Statistical analysis was performed using unpaired, two-tail Student’s *t*-test (^∗^*p* < 0.05; ^∗∗^*p* < 0.005; ^∗∗∗^*p* < 0.001) and results are given as mean ± SD (Results of three independent experiments). **(C)** GMCSF BMDC from C57BL/6 mice were infected with *B. abortus* 2308 (30:1). At 4, 8, 24, and 48 h post-infection, cells were recovered and RNA was extracted. Gene expression is represented as a fold increases between non-infected and infected cells. Statistical analyses were performed by using the comparative CT Method (ΔΔCT method) given by 2^-ΔΔCT^. The dotted line represents a fold increase of 2, the statistical significant threshold in this method. Results are given as mean ± SD (Results of three independent experiments).

We first measured the AA concentration in *B. abortus* infected moDC culture supernatants at 16 h post-infection. In parallel, other intracellular bacteria (*T. whipplei*, *C. burnetii*, *O. tsutsugamushi*) were tested. Only *B. abortus* and *O. tsutsugamushi* induced a significant AA production compared to non-stimulated moDC (**Figure 2A**).

AA can be metabolized by the COX pathway (Harizi et al., 2008). Thus, to determine the impact of *B. abortus* on this pathway, we analyzed the transcriptional profile of several key genes involved in the PG and leukotriene pathways in infected cells (**Figure 2B**). Compared to unstimulated cells, infected human moDC strongly expressed *PTGS2* (33 times higher) and in a lesser extend *PTGER2*, *PTGER4 and PTGDS* (1,7, 3,1, and 1,3 times higher, respectively) (**Figure 2B**, upper panel). On the contrary, none of the genes involved in the leukotriene pathway were up-regulated following *Brucella* infection (**Figure 2B**, lower panel).

We next investigated the expression of *Ptgs2* in murine GM-CSF-derived DC at 4, 8, 24, and 48 h post-infection. We observed that as in human DC, murine *Ptsg2* mRNA level was strongly up regulated. *Ptsg2* expression was time-dependent, reaching a peak at 24 h post-infection (229.4-fold up regulated compared to non-infected cells) (**Figure 2C**).

These results show that *B. abortus* infection is capable of stimulating the AA metabolic pathway and downstream PG pathway both in human and mice DC.

### Intradermal Infection Is Characterized by a Strong *Ifng* and *Ptgs2* Signature

We next investigated the involvement of the PTGS-2 pathway *in vivo* upon infection in mice. We have first compared three different *Brucella* strains (*B. abortus* 2308, *B. melitensis* 16M, and *B. suis* 1330) and their ability to colonize CLN at 8 and 15 days post-infection using the oral infection model we previously shown to induce a specific colonization of CLN (von Bargen et al., 2014). We avoided to use intraperitoneal inoculation since it is associated to systemic infection and recedes from *Brucella* physiological routes of infection or vaccination (Grilló et al., 2012). Although bacterial loads in CLN were very similar at any time point for all *Brucella* species (Supplementary Figure 1A), *B. melitensis* and *B. suis* induced a higher lymphadenopathy compared to *B. abortus* at 15 days post-infection suggesting an enhanced inflammatory response in the CLN (Supplementary Figure 1B). We then decided to continue this study with *B. melitensis* 16M.

**FIGURE 3:**
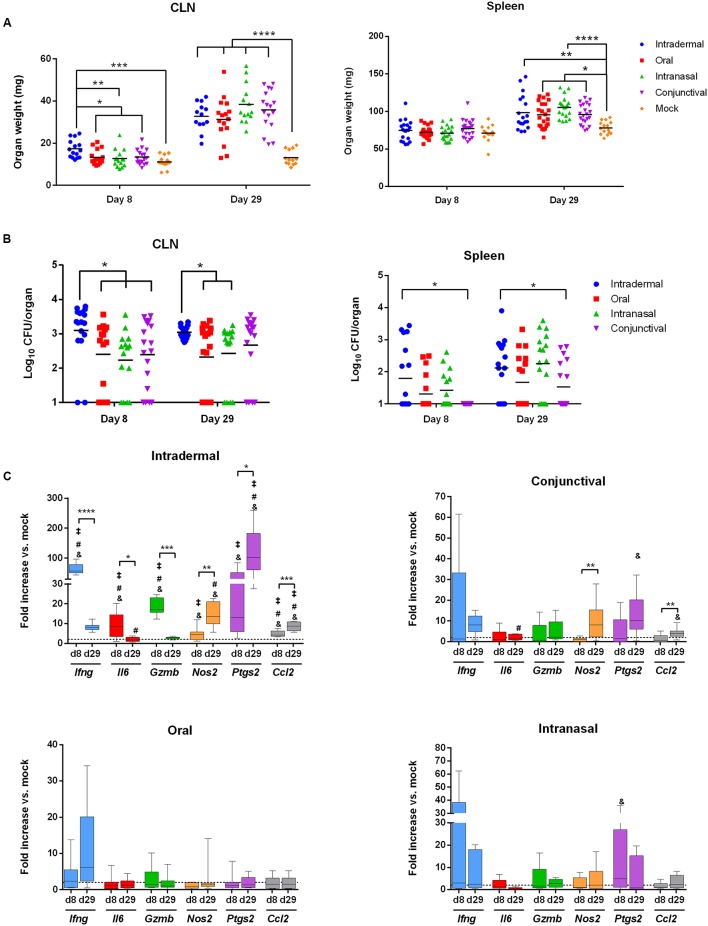
**The intradermal inoculation pathway induces a strong local inflammatory response in CLN**. C57BL/6 mice (*n* = 5 per group) were infected using the routes of infection described in the methods section. At 8 and 29 days post-infection, mice were sacrificed and organs were weighed and analyzed for their bacterial loads by plating homogenates on nutrient agar. Data represent mean of CLN (Left panel) and spleen (Right panel) weight **(A)** or CFU per organ **(B)** from three independent experiments. Analysis significance was determined using ANOVA (^∗^*p* < 0.05; ^∗∗^*p* < 0.005; ^∗∗∗^*p* < 0.001; ^∗∗∗∗^*p* < 0.0001). **(C)** C57BL/6 mice (*n* = 5 per group) were infected using different routes of infection as described in methods section. At 8 and 29 days post-infection, total RNA of the CLN was extracted and analyzed for inflammatory response gene expression by reverse transcription real-time PCR. Results are given as fold increase compared to the signal obtained for mock-infected mice. Statistical significance was determined using unpaired, two-tailed Student’s *t*-test (Results of three independent experiments) (^∗^*p* < 0.05; ^∗∗^*p* < 0.005; ^∗∗∗^*p* < 0.001; ^∗∗∗∗^*p* < 0.0001) and ^&^significant compared to the oral route; ^#^significant compared to the intranasal route and ^**‡**^significant compared to the conjunctival route using ANOVA.

WT C57BL/6 mice were inoculated with *B. melitensis* by oral administration (with 10^9^ bacteria per mouse) as previously described (von Bargen et al., 2014) or by intranasal (with 10^5^ bacteria per mice), conjunctival (10^9^ bacteria per mice) or intradermal (10^4^ bacteria per mice) routes. Mice were sacrificed at 8 and 29 days post-infection and the weight and bacterial load of CLN and spleens were measured (**Figures 3A,B**).

At 8 days post-infection, the intradermal route was the only route capable of inducing lymphadenopathy compared to mock-treated mice and to other inoculation methods (**Figure 3A**, Left panel). No splenomegaly was observed at 8 days post-infection compared to mock-treated mice (**Figure 3A**, Right Panel). These results correlated with bacterial loads. CFU numbers in CLN were higher in mice infected intradermally compared to the other infection routes (**Figure 3B**, Left panel). However, spleens were poorly colonized as observed in all infection conditions (**Figure 3B**, Right panel). At 29 days post-infection, all inoculation routes resulted in lymphadenopathy and induced splenomegaly (**Figure 3A**). The increase in CLN and spleen weight was not associated with an increase in bacterial load (**Figure 3B**). This suggests the establishment of an inflammatory response in these organs.

We next investigated the local immune response induced by *Brucella* infection in the spleens and CLN by extracting mRNA at 8 and 29 days post-infection. Results were analyzed as a fold increase compared to their mock counterparts. No significant response was observed in the spleen for any infection routes (Supplementary Figure 2). On the contrary, a clear inflammatory response was observed in the CLN of mice infected intradermally from 8 days post-infection onwards (**Figure 3C**). This response led to a strong up-regulation of *Ifng*, *Gzmb* and *Ptgs2* mRNAs (64, 18.6, and 30.8-fold, respectively) (**Figure 3C**). At 29 days post-infection, *Ifng*, *Gzmb*, and *Il6* mRNA levels strongly decreased whereas a strong up-regulation of *Ptgs2* and in a lesser extent *Nos2* and *Ccl2* mRNA levels remained (**Figure 3C**). The inflammatory response was less pronounced using the other inoculation routes. Indeed, no significant changes between day 8 and 29 were observed in terms of mRNA expression levels except for conjunctival inoculation displaying an increase of *Nos2* and *Ccl2* mRNA levels (Fold change: 10.3 and 2.9, respectively) (**Figure 3C**).

In conclusion, the intradermal infection route is the most potent at inducing inflammatory genes expression and *Ptgs2* seems to be a robust marker of *Brucella* intradermal infection compared to intranasal, conjunctival or oral infection routes. At 29 days post-infection following *B. melitensis* intradermal inoculation, the expression of *Il6* was significantly higher compared to intranasal infection. *Nos2* (compared to the oral and intranasal routes), *Ptgs2* and *Ccl2* genes (compared to all the other routes) were also increased (**Figure 3C**). Interestingly, no systemic response was observed in mice sera at any time independently from the inoculation route except for IFN-γ after intradermal infection. However, low but significant amounts of TNF-α were detected in the sera of mice infected intradermally at 8 days post-infection (Supplementary Figure 3).

Overall, these results strongly suggest that in the CLN after intradermal infection a robust inflammatory response characterized by an *Ifng* and a *Ptgs2* signature takes place.

### COX-2 Inhibition Reduces Bacterial Burden *In vivo*

After having identified that the intradermal route was the more potent to induce *Ptgs2* expression during *B. melitensis* infection, we evaluated *in vivo* the involvement of COX-2 during infection. WT C57BL/6 mice were challenged intradermally with *B. melitensis* (10^4^ bacteria per mice) and were given NS-398, a specific COX-2 inhibitor, or DMSO vehicle (mock control) by intraperitoneal administration daily for 7 days.

**FIGURE 4:**
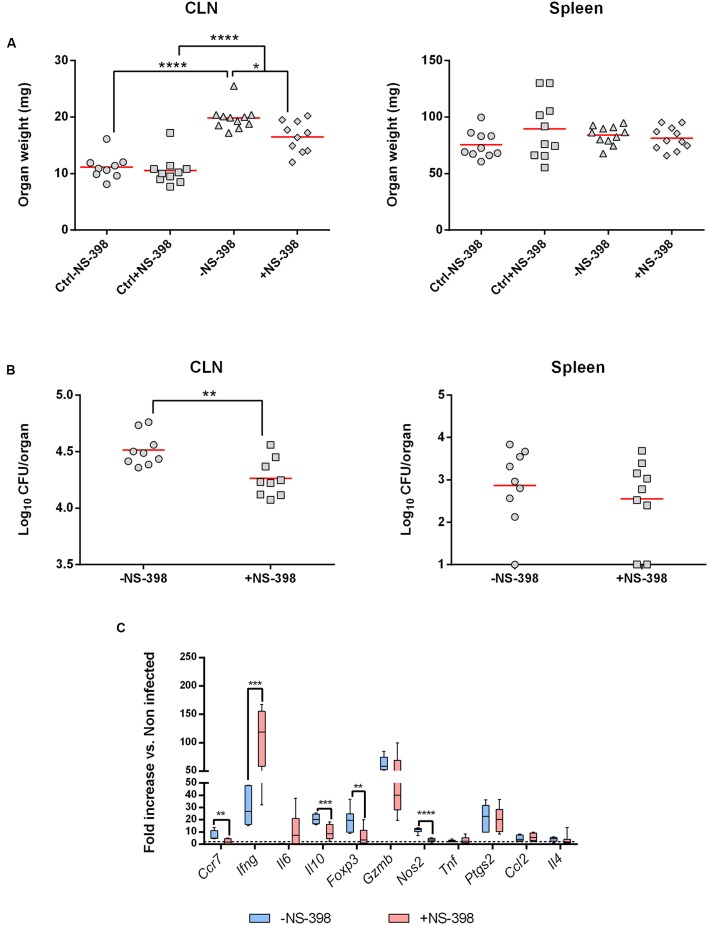
**Cyclooxygenase-2 inhibition induces a decrease of bacterial burden in draining lymph nodes**. C57BL/6 mice (*n* = 5) were infected with *B. melitensis* (10^4^ bacteria per mice) intradermally into ear pinnae. After infection mice received 15 mg/kg of COX-2 inhibitor (NS-398) or DMSO (mock-treated mice) intraperitoneally daily for 7 days. Data represent two independent experiments. **(A)** At 8 days post-infection mice were sacrificed and organs were harvested and CLN (Left panel) and spleen (Right panel) were weighted. Statistical significance was determined using ANOVA (^∗^*p* < 0.05; ^∗∗∗∗^*p* < 0.0001). **(B)** At 8 days post-infection mice were sacrificed and organs were harvested and analyzed for their CLN (Left panel) or spleen (Right panel) bacterial loads by plating homogenates on nutrient agar. Statistical significance was determined using unpaired, two-tailed Student’s *t*-test (^∗∗^*p* < 0.005). **(C)** At 8 days post-infection, total RNA of the CLN was extracted and analyzed for expression of genes involved in inflammatory response by reverse transcription real-time PCR. Results are given as fold increase compared to the signal obtained for mock-infected mice. Statistical significance was determined using unpaired, two-tailed Student’s *t*-test (^∗∗^*p* < 0.005; ^∗∗∗^*p* < 0.001; ^∗∗∗∗^*p* < 0.0001).

We first looked at CLN and spleen weights at 8 days post-infection. Independently of the treatment, infected mice presented a lymphadenopathy compared to uninfected mice. However, NS-398 treatment induced a decrease in CLN weight compared to non-treated infected mice (**Figure 4A**, Left panel). This effect was consistent with the anti-inflammatory properties of NS-398. No significant effect could be observed in the spleen (**Figure 4A**, Right panel). This response was accompanied by a decrease of CFU numbers in CLN in treated mice (**Figure 4A**, Left panel) and no significant response was observed in the spleen (**Figure 4B**, Right Panel).

The local inflammatory response in NS-398-treated mice was characterized by a strong decrease of *Il10* and *Nos2* expression and in a lesser extent of *Ccr7* and *Foxp3* compared to untreated mice. However, *Ifng* mRNA level strongly increased following NS-398 treatment. All the other genes tested did not exhibit any significant changes in their expression (**Figure 4C**).

Altogether, these results indicate that inhibition of COX-2 decreases bacterial load in CLN by acting on *Il10 and Ifng* expression levels.

## Discussion

In this study, we investigated the impact of *Brucella* spp. infection on the COX-2 metabolic pathway. We first assessed the ability of *B. abortus* to stimulate AA production in DC. We also confirmed using a genomic approach that PG pathway downstream partners were overexpressed. We observed that *B. abortus* triggers a higher level of AA in human moDC infected with *B. abortus* compared to cells infected with other intracellular bacteria (*C. burnetii, T. whipplei, O. tsutsugamushi*). This response was accompanied by an increase of *Ptgs2* mRNA levels in both human and murine DC. Interestingly, we did not detect any major change in the expression profile of the leukotriene pathway and specifically for *ALOX5*, the gene encoding for the 5-LO in humans. This result is contradictory with previously published data showing that 5-LO was strongly up-regulated in *Brucella* infected spleens as early as one-week post-infection in mice and was accompanied by leukotriene B_4_ and lipoxin A_4_ production (Fahel et al., 2015). This discrepancy may be explained by the *in vitro* DC model used in our study compared to total splenocytes used in the study published in 2015 and also possible differences between the mouse and human models. Moreover, cells expressing 5-LO have not yet been identified *in vivo*, thus, it could be possible that DC are not the source of leukotrienes during *B. abortus* infection.

Transcriptional profiling showed an increase of *PTGER2*, *PTGER4*, and *PTGSD* expression in infected human moDC compared to unstimulated cells. Interestingly, it has been reported that PGE_2_ can stimulate IL-10 secretion by mature BMDC leading to a decrease of IL-12 secretion. This effect is dependent on the expression of EP2/EP4 receptors encoded by *Ptger2* and *Ptger4*, respectively (Harizi et al., 2003). Similarly, macrophage treatments with PG analogs stimulating the EP2/EP4 receptors lead to an increase of IL-10 secretion and concomitantly a decrease of TNF-α secretion (Shinomiya et al., 2001). Moreover, in a *Pseudomonas aeruginosa* intranasal infection model, this immunomodulatory effect towards IL-10 secretion seems to be mediated by EP2 since EP2^-/-^ mice presented a lower bacterial burden in the lung (Sadikot et al., 2007). Considering the negative impact of IL-10 on the establishment of a protective immune response during brucellosis, PG pathway could contribute to bacterial persistence.

Then, we determined the levels of expression of *Ptgs2 in vivo* using different infection routes. For this purpose, we chose to study physiologically relevant inoculation routes such as nasal, oral, and conjunctival infection routes that mimic natural exposure to bacteria. We also included the intradermal infection route often used for vaccination (Kim et al., 2012). We focused on CLN since CLN also drains the eye (Hamrah and Dana, 2007) and the nasal mucosa (Wolvers et al., 1999) and we previously shown that this organ was preferentially targeted by *Brucella* after oral inoculation (von Bargen et al., 2014). COX-2 being important during inflammatory process, contrary to the *in vitro* experiments, we decided to use *B. melitensis* as it seems that this species induced an enhanced inflammation in CLN compared to *B. abortus*.

As anticipated, we observed a tropism of bacteria for the CLN and a marked lymphadenopathy compared to mock-treated mice as soon as 8 days post-infection in mice infected intradermally and in all conditions at 29 days post-infection. This effect was particularly marked when using the intradermal route. When looking at the local immune response in CLN, we observed that the intradermal route induced a particular gene signature characterized by a strong up-regulation of *Ifng* and *Ptgs2* mRNA levels compared to the other tested routes. However, we cannot exclude a difference in terms of kinetics due to the fact that intradermal infection delivers directly the bacteria into the tissue. On the contrary, oral, intranasal, and conjunctival inoculations expose bacteria to physiological barriers such as mucosal surfaces that can delay the bacterial trafficking to the draining lymph nodes. Indeed, we observed that at 8 days post-infection bacterial load was significantly higher after intradermal infection but this difference tends to be reduced at later time points. Moreover, using a *Yersinia pestis* intradermal infection model it has been shown that bacterial dissemination does not involve bacteria active transport through phagocytes to the draining lymph nodes (Gonzalez et al., 2015). At the beginning of the chronic phase (29 days post-infection), we observed a strong decrease of *Ifng, Il6, Gzmb* concomitant with and an increase of *Nos2*, *Ptgs2* mRNA levels. Interestingly, it has recently been shown that synergic induction of COX-2 by IFN-γ and TNF-α limits type-1 immune response in tumor microenvironment by concomitant action of IL-10, NOS2, and Indoleamine 2,3-dioxygenase (Wong et al., 2016). Thus, we can hypothesize that the induction of *Ptgs2* is beneficial for the bacteria.

We then used a specific COX-2 inhibitor (NS-398) and demonstrated the beneficial potential of such an inhibition in favor of the host. Indeed, COX-2 inhibition correlated with a decrease in bacterial load in CLN concomitant with a decrease of *Ccr7*, *Il10*, *Foxp3*, *Nos2* mRNA levels and an increase of *Ifng* expression. At early time points, the induction of *Ptgs2* gene expression could be used by the bacteria to stimulate not only their replication but also the migration of the infected cells to other secondary organs as suggested by the effect on *Ccr7* expression. It is also known that Th1 immune response characterized by IFN-γ and IL-12 secretion is important to control infection and that production of IL-10 has a negative impact in the course of brucellosis control by the host (Zhan and Cheers, 1995; Brandao et al., 2012). Then, we can hypothesize that the beneficial effect observed in the presence of COX-2 inhibitor treatment can be associated with a strong Th1 response. Moreover, it has been demonstrated that in absence of IL-10, mice are more resistant to *Brucella* infection and this effect is correlated with an increase of pro-inflammatory cytokine secretion (Corsetti et al., 2013). The decrease of *Il10* mRNA levels is also accompanied by a decrease of *Foxp3* expression, a marker of regulatory T lymphocytes (T-reg). After intraperitoneal infection, it has been shown that CD4^+^CD25^+^ T cells produce IL-10 during acute brucellosis that favor bacterial persistence in mice (Xavier et al., 2013). CD4^+^CD25^+^ T cell population in parotid and retropharyngeal lymph nodes has been shown to increase following conjunctival infection in sheep (Suraud et al., 2007). CD4^+^CD25^+^ T cells with regulatory activity express the transcription factor Foxp3 (Hori et al., 2003). Interestingly, following NS-398 treatment, we observed a significant decrease of *Foxp3* expression. This finding is in accordance with the decrease of *Il10* expression. Thus, we hypothesize that infection stimulates regulatory/suppressor T-cells that can favor bacterial persistence through the secretion of IL-10. A similar effect has been described during *Mycobacterium tuberculosis* infection where COX-2/PGE_2_ axis stimulates T-reg expansion (Garg et al., 2008; Holla et al., 2016). Thereby, T-reg accumulation contributes to the decrease of bacterial clearance in infected mice (Kursar et al., 2007). During *Francisella tularensis* live vaccine strain intranasal infection, the inhibition of the COX-1 and COX-2 increased the number of IFN-γ producing T lymphocytes in the lung leading to a decrease of the bacterial load (Woolard et al., 2008). This is correlated with our observations showing that COX-2 inhibition leads to an increase of *Ifng* mRNA levels in CLN.

Our results corroborate other studies which have investigated the involvement of the COX-2 pathway during bacterial infection by *Pseudomonas aeruginosa* (Sadikot et al., 2007), *Burkholderia pseudomallei* (Asakrah et al., 2013), and *Streptococcus pyogenes* (Goldmann et al., 2010). COX-2 inhibition translates into a decrease of bacterial burden in CLN. It would be interesting to look at later time points to know whether or not COX-2 inhibition can cause a bacterial clearance. Taken together, our results suggest that *Brucella* has taken advantage of the PG pathway to survive and replicate in host cells and that COX-2 inhibition is not only crucial to control brucellosis but also other bacterial infectious diseases.

## Author Contributions

AG, J-PG, and J-LM conceived and designed the experiments. AG, LG, AP, and KVB performed the experiments. AG, LG, and J-PG analyzed the data. AG and J-PG wrote the paper.

## Conflict of Interest Statement

The authors declare that the research was conducted in the absence of any commercial or financial relationships that could be construed as a potential conflict of interest.

## Abbreviations

5-LO: 5-Lipoxygenase
AA: Arachidonic Acid
BMDC: Bone marrow-derived dendritic cell
Ccl2: C-C-chemokine ligand 2
Ccr7: C-C-chemokine receptor 7
CFU: Colony forming units
CLN: Cervical lymph nodes
COX-2: Cyclooxygenase-2
DC: Dendritic cells
DMSO: Dimethyl sulfoxide
EP: Prostaglandin E receptor
Foxp3: Forkhead box 3
GM-CSF: Granulocyte macrophage colony-stimulating factor
Gzmb: Granzyme B
IFN: Interferon
IL: Interleukin
LPS: Lipopolysaccharide
moDC: Monocyte derived dendritic cell
MOI: Multiplicity of infection
NOS_2_: Nitric oxide synthase 2
NS-398: *N*-[2- (cyclohexyloxy)-4-nitrophenyl]-methanesulfonamide
PBMC: Peripheral blood mononuclear cells
PG: Prostaglandins
PTGDS: Prostaglandin D synthase
PTGER: Prostaglandin E receptor
Ptgs2: Prostaglandin-endoperoxide synthase 2
Th1: T helper 1
TLR: Toll-like receptor
TNF-α: Tumor Necrosis Factor α
TSA: Tryptic Soy Agar
TSB: Tryptic Soy Broth
