# Supplementary material for: COX-2 Inhibition Reduces Brucella Bacterial Burden in Draining Lymph Nodes
Source: Front Microbiol. 2016 Dec 12;7:1987. doi: 10.3389/fmicb.2016.01987 (PMC5149544; doi:10.3389/fmicb.2016.01987)
Supplement: Supplementary file 1 [file Image_1.PDF]

Supplementary Material

COX-2 INHIBITION INDUCES A PROTECTIVE RESPONSE AGAINST *BRUCELLA* INFECTION IN VIVO

Aurélie Gagnaire[1], Laurent Gorvel[2], Alexia Papadopoulos[1], Kristine Von Bargen[1], Jean-Louis Mège[3] & Jean-Pierre Gorvel[1*]

*** Correspondence:** Corresponding Author: gorvel@ciml.univ-mrs.fr

1 Supplementary Data

Supplementary Material should be uploaded separately on submission. Please include any supplementary data, figures and/or tables.

Supplementary material is not typeset so please ensure that all information is clearly presented, the appropriate caption is included in the file and not in the manuscript, and that the style conforms to the rest of the article.

2 Supplementary Figures and Tables

For more information on Supplementary Material and for details on the different file types accepted, please see here.

2.1 Supplementary Figures

Supplementary 1

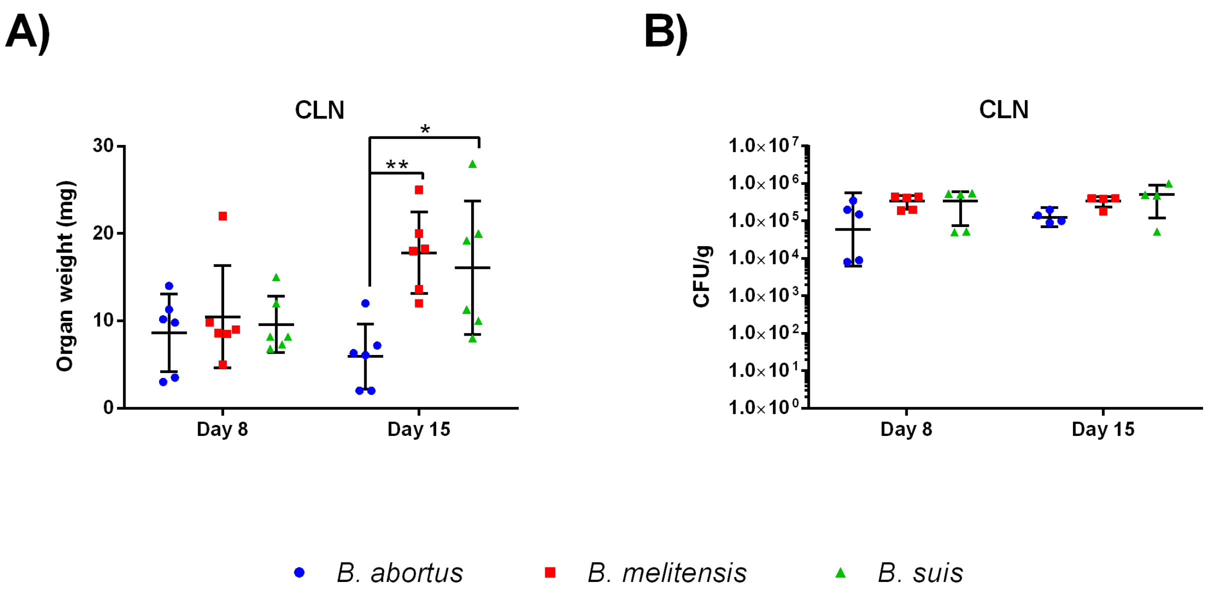

Supplementary Figure 1 (SI1).

Supplementary 2

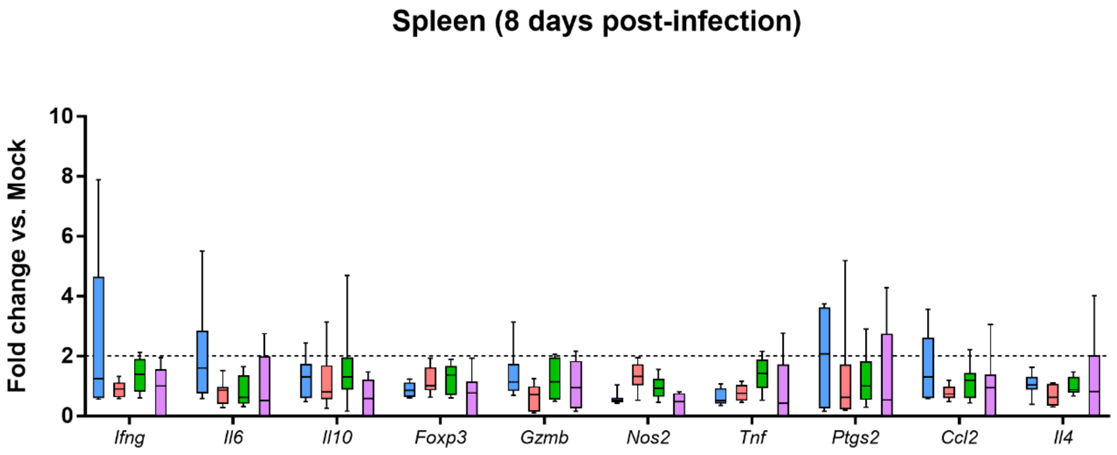

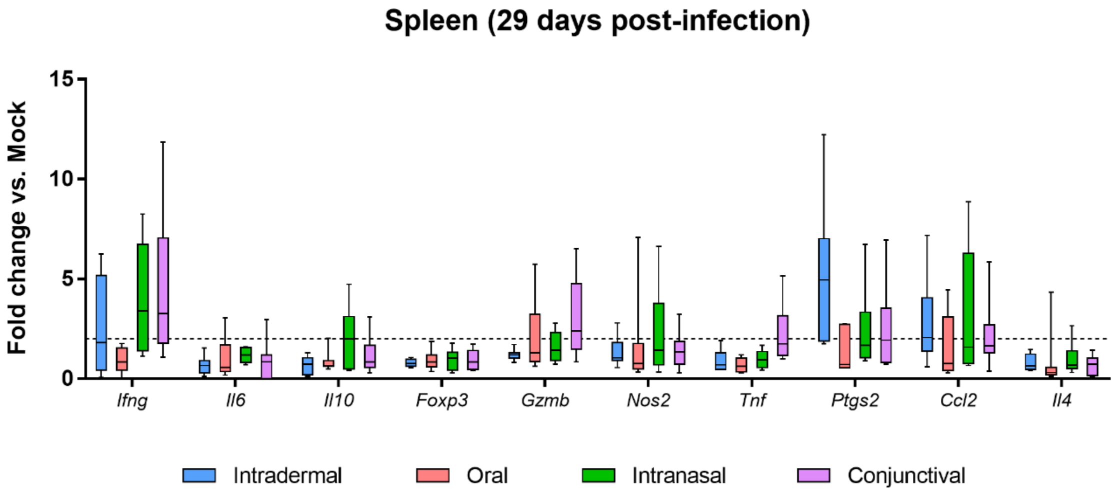

Supplementary Figure 2 (SI2).

Supplementary 3

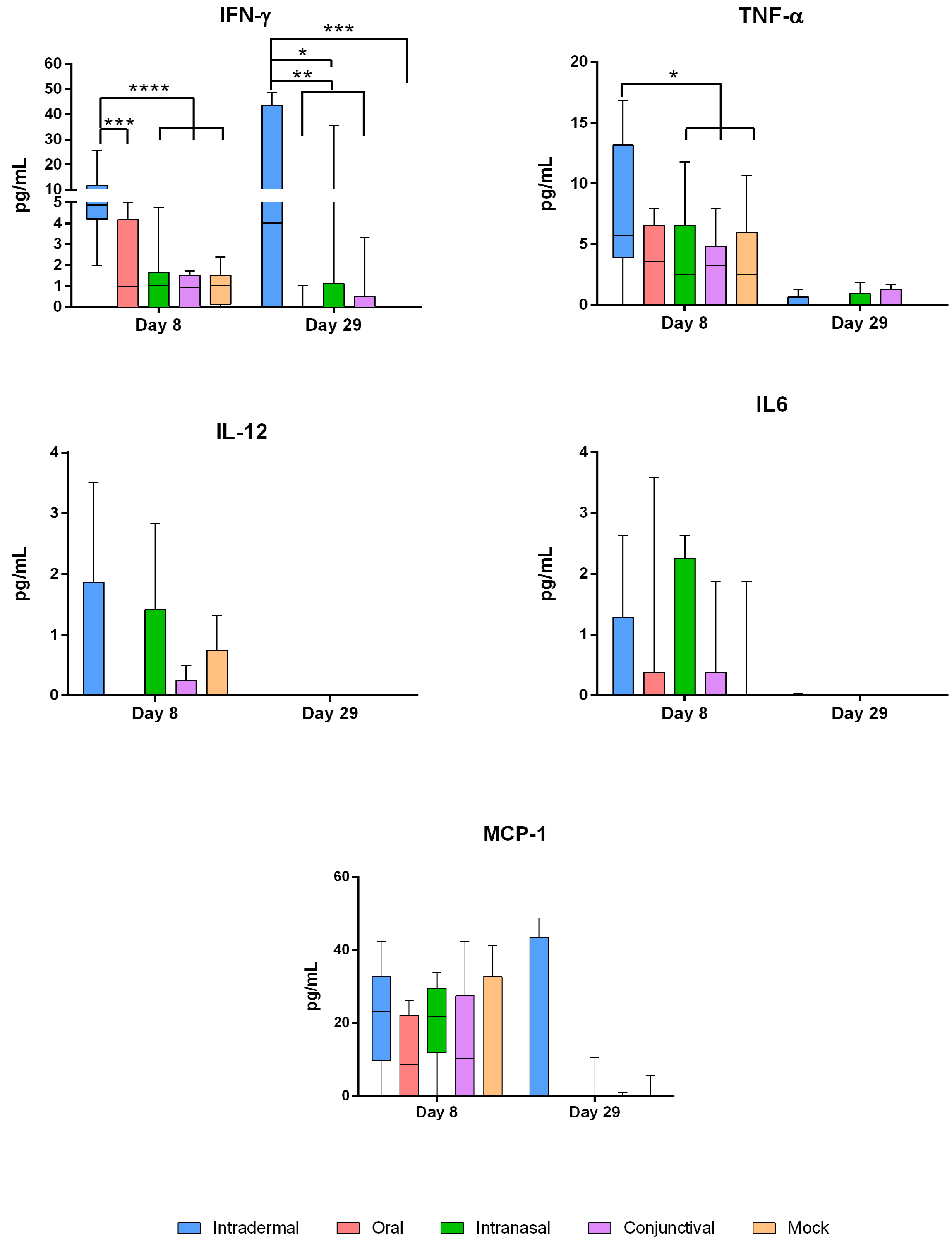

Supplementary Figure 3 (SI3).

Supplemental information files

SI1 Oral bacterial challenge with different *Brucella* strains induces the same level of bacterial colonization of CLN. (A) C57BL/6 mice (n=6) were infected by the oral route with *Brucella abortus* smooth virulent strain 2308, *Brucella melitensis* 16M or *Brucella suis* 1330 using 10^9 bacteria per mice. At 8 and 15 days post-infection the CLN were harvested and weighted. Statistical significance was determined using ANOVA (*p <0.05; **p <0.005). **(B)** C57BL/6 mice (n=6) were infected by the oral route with *Brucella abortus* smooth virulent strain 2308, *Brucella melitensis* 16M or *Brucella suis* using 10^9 bacteria per mice. At 8 and 15 days post-infection the CLN were harvested and analysed for their bacterial load by plating homogenates on nutrient agar. Results are given as mean ± SD.

SI2 Infection does not induce significant pro-inflammatory genes expression in spleen compared to mock-treated mice. C57BL/6 mice (n=5 per group) were infected using different routes of infection as described in methods section. At 8 and 29 days post-infection, total RNA of the spleen was extracted and analyzed for expression of genes involved in inflammatory response by reverse transcription real-time PCR. Results are given as fold increase compared to the signal obtained for mock-infected mice. Statistical analysis was performed by using the comparative CT Method (ΔΔCT method) given by $2^{-\Delta\Delta CT}$. The dotted line represents a fold increase of 2, the statistical significant threshold in this method (Results of 3 independent experiments).

SI3 Only the intradermal infection route results in significant low secretion of IFN-γ and TNF-α in blood compared to the other infection routes. C57BL/6 mice were infected with 10^9 (oral and conjunctival routes), 10^5 (intranasal route) or 10^4 (intradermal route) *B. melitensis*. At 8 and 29 post-infection, blood of mice was recovered and analyzed for the presence of cytokines. Data represent the results of two independent experiments with 5 mice per group. Statistical significance was determined using ANOVA (*p <0.05; **p <0.005; ***p <0.001; ****p <0.0001). Results are given as mean ± SD.
